# *‘Mummy told me that I have HIV*, *that is the only thing she told me’*: Experiences of HIV status disclosure to children in Masaka Region, Uganda

**DOI:** 10.1371/journal.pone.0285990

**Published:** 2023-05-24

**Authors:** Robert Kairania, Washington Onyango-Ouma, Tom G. Ondicho, Joseph Kagaayi, Godfrey Kigozi

**Affiliations:** 1 Department of Anthropology, Gender and African Studies, University of Nairobi, Nairobi, Kenya; 2 Rakai Heath Sciences Program, Uganda Virus Research Institute, Entebbe, Uganda; PhD, PLOS, UNITED KINGDOM

## Abstract

**Background:**

In sub-Saharan Africa (SSA), disclosure of HIV status to children remains low. Few studies have examined how children navigate and come to terms with their HIV status. The aim of this study was to explore experiences of children about disclosure of their HIV status.

**Methods:**

Between October 2020 and July 2021, 18 purposively selected children aged between 12–17 whose HIV status had been disclosed to them by their caregivers or healthcare providers (HCPs) were recruited for this study. We conducted 18 in-depth interviews (IDIs) to collect data for this study. Data were analyzed using the semantic thematic analysis approach.

**Results:**

Primary data obtained through IDIs revealed that disclosure of HIV status to children occurred as a one-time event without pre—disclosure preparatory planning or focused post disclosure follow-up counseling irrespective of the discloser. Post disclosure psycho-social experiences elicited mixed responses. Some children experienced insults and belittlement and stigma and discrimination in the family and community for out-of-school children and at school for school-going children. Positive disclosure experiences involved receiving support to improve ART adherence through constantly being reminded to take their medications timely at the workplace, by supervisors, for working children and by teachers, at school for school-going children.

**Conclusions:**

This research contributes to knowledge about children’ s experiences of being HIV infected and can specifically be used to improve disclosure strategies.

## 1. Introduction

Many children infected with HIV perinatally are now living or surviving into adolescence and early adulthood [1]. The number of children, 0–14 years living with HIV in the world increased from 2,500,000 in 2009 [2] to 2,800,000 in 2018 [3]. Sub-Saharan Africa (SSA) accounts for ninety percent of them [4]. In Uganda, about 95, 000 children 0–14 years are living with HIV [5] and 10% (9735/95,000) of these children are from Maska region [6]. The survival of these children is, largely, a result of free access to Anti-Retroviral Therapy (ART) by the support of the United States through the President’s Emergency Plan for AIDS Relief (PEPFAR), the United Nations International Children Emergency Fund (UNICEF), the Global Fund and the World Health Organization (WHO) among others. For example, the number of children 0–14 years accessing ART, in Uganda, increased from 43,803 in 2014 [7] to 66, 203 in 2019 [8]. In Masaka region, 6815 children are on ART out of the estimated 9735 children living with HIV [6]. Although there is still a gap in ART access, the impact of using ART has reduced HIV/AIDS related deaths in the country from 17,000 in 2010 to 4, 800 in 2019 among children in the country [8]. ART have not only reduced HIV/AIDS related morbidity and mortality among children, but HIV is also moving from a fatal disease to a chronic manageable health condition [9]. Chronic disease management emphasizes self-care combined with biomedical disease management to maintain a normal life [10, 11]. Disclosure of HIV status is one of the first steps towards the realization of management of HIV as a chronic disease in children living with HIV. Research shows that in SSA disclosure of HIV status to children is low ranging from 1.2% to 72% [12–16]; in Kenya it varies from 1.7%-26%[17]; in Tanzania it ranges between 22%-33% [18] and in Uganda it varies from 29% - 43% [19]. Culturally sanctioned gender norms, religion and religiosity have been blamed for low rates of disclosure [20] because they have shown to entrench silence and secrecy [21]. The statistics are based on HIV disclosure that occurs between the ages of 6 and 17 years [17–19, 22] as a one-time event [22, 23] with no pre-and post-disclosure follow-up counselling with children about HIV [22, 24] because their caregivers lack knowledge on the process of disclosure to support the children [25]. Some children are told lies regarding their HIV status by caregivers [14, 24, 26–28] for fear of children reacting negatively to the disclosure [29–31], concerns of negative social consequences such as stigma [25] or fear of blame for infecting the children [32, 33].

In Uganda, although the national policy guidelines require that children perinatally infected with HIV be told about their status by the age of 10 years [34], this reality is a rarity [19, 28]. This is partly because healthcare providers (HCPs) lack disclosure skills [35], time, and commonly relegate HIV disclosure responsibility to caregivers and parents [34]. This has complicated the disclosure process leading to few children above 10 years learning their HIV status [28]. HIV disclosure to children is still being done by HCPs and caregivers who have not been trained or equipped with the requisite skills and knowhow to deliver developmentally age-appropriate disclosure to children about their HIV status [35–37]. Some children receive HIV disclosure in terrifying, inappropriate, and in life-disrupting moments [37, 38]. When children get to know their HIV status without adequate psychological/mental preparation, it becomes difficult to prevent the psychological distress that comes from such haphazard disclosure events [39]. Thus, it is important that we understand the experiences of children learning their HIV status and the post disclosure meanings and implications in the real world to offer appropriate help and support. We explored the experiences of disclosure of HIV status to children taking daily ART in the Masaka region of Uganda

## 2. Materials and methods

### 2.1 Study setting

The study was conducted in Masaka region between October 2020 and July 2021. Masaka region is located about 125 kilometers south- west of Kampala capital city, in the southern part of Uganda. It is where the first HIV case in Uganda was identified in 1982 [40]. Masaka region has consistently had the highest HIV burden in the country according to data from three consecutive national HIV/AIDS surveys at: 10.6% in 2011, 8.0% in 2016 and 8.1% prevalence in 2020 respectively [5, 41, 42]. This is probably because Masaka region has the largest number of Lake Victoria fishing communities in the country. The fishing communities of Lake Victoria are traditionally known to have disproportionate HIV prevalence and currently stands at 18% [43] with a 5–6% HIV prevalence among adolescents aged 13–19 years infected mainly through the mother-to-child transmission mode [44].

### 2.2 Study design and informants

This study is part of a larger explanatory sequential mixed-methods research project with the goal of investigating the trajectories of disclosure of HIV status to children and their adherence to ART. The study employed a cross-sectional qualitative design by utilizing in-depth Interviews (IDIs) with children who were aware of their HIV status from an earlier cross-sectional quantitative study whose findings have been reported elsewhere [45]. Qualitative design was chosen to explore children’s detailed accounts of life experiences, meanings, and concepts on disclosure experiences [46] and thus extending earlier quantitative findings. The study purposively selected 18 HIV infected children identified via the quantitative sub-study component of a larger explanatory sequential mixed-methods research project where they revealed that they were aware of their HIV infection status following disclosure either by an HCP or a caregiver and were taking daily ART. Children were aged 12–17 years, enrolled in both rural and urban health facilities of Masaka region. The rural and urban facilities were representative of size of the ART units and variation in the client load and represented the diversity of the HIV prevalence, understanding and context.

### 2.3 Data collection

Data were collected through IDIs with assenting children, to explore their experiences with receipt of disclosure of HIV status guided by the semi-structured interview guides. Caregivers were phone-called or home-visited two days, prior, to make appointments for the interviews with the children. The interviews were conducted in Luganda language, using a semi-structured interview schedule which permitted the interviewer to make probes and ask follow-up questions. All the interviews were conducted in a secure and private location within the informant’s village and were audio-recorded lasting about 60 to 90 minutes. Information on schooling, who provided the disclosure, where the disclosure happened, situations surrounding /timing of the disclosure, content of the disclosure, reactions to the disclosure, social support and sources of that support following disclosure, self-disclosure to others, among others was collected from the IDIs. Interviews were transcribed verbatim in Luganda language and back translated into English before analysis. Data security was maintained by storing all data files on password protected computers. We used alphanumeric codes to identify participants’ responses to maintain the anonymity of study participants. Informed consent documents and audio recordings were also stored in secure settings.

### 2.4 Data analysis

Audio-recorded interviews and field notes were transcribed into Microsoft Word. This was followed by a rigorous systematic reading and rereading of the transcripts. We used thematic analysis to manually analyze the data guided by Braun & Clarke [47] basic thematic analysis steps. We did not use any data analysis software. By use of a highlighter, data was coded to identify segments in the data that indicated potential patterns linked to the research question and conceptual framework. Theme code sheets in Microsoft excel were developed wherein specific codes were named and defined. Codes were sorted and organized into potential semantic themes and semantic themes combined to form overarching themes. A table was drawn to visualize the relationship between codes, between themes, and between different levels of themes (main overarching themes and sub-themes within them). Some initial codes went on to form main themes, whereas others formed sub-themes. We then considered the validity of each individual theme in relation to the data set and how they fitted together to tell an overall story about the data as a whole in relation to answering the research question to build valid arguments to summarize the findings. Data were triangulated through making comparisons between the field notes, tape recorded semi-structured interviews and photographs (where informants permitted). We also kept an audit trail of the research processes and procedures from inception to realize trustworthiness.

### 2.5 Ethical considerations

The study received ethical approval from the Uganda Virus Research Institute-Research Ethics Committee (UVRI-REC) (Ref# GC/127/19/12/757). Final approval to conduct the study was obtained from the Uganda National Council for Science and Technology (UNCST) (research registration number HS522ES). The study protocol was also approved by the Faculty Post-Graduate Studies Committee at the Institute of Anthropology, Gender, and African Studies, University of Nairobi, after giving a seminar on 14^th^ March 2019. Informed consent was obtained from caregivers of sampled children and informed assent from the children themselves. Both the caregivers and children provided written informed consent or assent by signing or thumbprinting pre-prepared consent forms. The children and their caregivers were given detailed information about the study objectives, were reassured about their privacy and confidentiality of the information collected from them, and that participation was voluntary. They were then asked for their informed consent and assent. Data were identified by study numbers but not names to maximize confidentiality.

## 3. Results

### 3.1 Characteristics of informants

18 informants aged 12–17 years, with a mean age of 16 years participated in this study. Seven of the 18 children were in school and 11 were out of school. Of the children who had dropped out of school, 10 were involved in growing crops for a livelihood and one was employed as a maid. At the time of the study, among the out of school informants, four informants were living with biological mothers, one with a biological father, one informant was living with both biological mother and father in the same household, three with maternal aunts, one (employed as a maid) was living with a superior while one (female) had gotten married. Of the informants in school, four were in boarding section and three were commuting daily to attend school. In terms of receipt of disclosure of HIV status, 13 children received disclosure from HCPs at the HIV clinics in facilities and five children received disclosure from caregivers at home.

### 3.2 Themes and subthemes

Analysis of transcripts revealed five main themes: disclosure circumstances, disclosure process, reactions to disclosure, post-disclosure psychosocial experiences, and self-disclosure to others. The transcripts also unveiled several subthemes regarding HIV disclosure experiences of children taking ART in Masaka region. These themes and subthemes are summarized in Table 1 below.

**Table 1:** Summary of themes and sub-themes.

Theme	Sub-Theme	Sample of Codes
Disclosure circumstances	Poor Adherence to ART	Unstable adherence
		Uneven medication taking
		Adherence trend
	Endless questioning about continued medications	Insisting to be told the truth
		Why only me taking medications
	Refusal to take medications	Not experiencing any pain
		Throwing medicines
	Intermitted illnesses	On and off fever
		Wounds allover
Disclosure process	Disclosure preparation	Death threats
		High viral load
	Disclosure event	Born with HIV
		Infected with HIV
Reactions to disclosure	Emotional distress	Feelings of sickness and confusion
		Feelings of nausea and vomiting
	Anger and Irritation	Felt irritated
		Became angry
		Why me alone
Post disclosure psychosocial experiences	Stigma and discrimination	Acts of rejection
	Insults and belittlements	Bullying from colleagues
		Walking dead body
	Support from peers and community members	Adherence support
		Strong encouragement
Self-disclosure to others	Negatives of disclosure	Must keep it by yourself
		Spreading rumours
		Less important
	Reminders about medication time	Managing medication time
		Prompts each other
	Sexual relationships	Continued getting along
		Partner acceptance
		Supporting HIV clinic attendance

The themes and subthemes are expounded below.

#### 3.2.1 Disclosure circumstances

This theme represents circumstances and situations under which HIV disclosure occurred among children in this study. Four subthemes were identified under this theme: poor adherence to ART, endless questioning about continued medications, refusal to take medications and intermittent illnesses and each of these is described below.

*3*.*2*.*1*.*1 Poor Adherence to ART*. Children narrated that they were told about their HIV status in circumstances when their adherence trend to HIV treatment had deteriorated, were unstable on treatment or were taking medications intermittently. This compelled caregivers or healthcare providers to provide HIV disclosure to the children as illustrated by the following four informants:

*At that time*, *my adherence to medication*, *[antiretroviral treatment] was not good*, *and then the healthcare provider disclosed to me that I am HIV positive* (*Male informant; 15 years old; out of school*).

*For me by the time I came to know it*, *………is when I had a poor adherence trend*, *and the healthcare provider told me that I was to die because I had HIV infection with a high viral load (Male informant; 15 years; in school*).

*Whenever I could take medicine for one month and feel good*, *the following month I could not take it*. *I could only take it when I feel bad again (Female informant; 17 years old; out of school*).

*Right now*, *I am on second line [ART regimen] because I was unstable in adherence (male informant; 17 years; in school*).

*3*.*2*.*1*.*2 Endless Questioning about Continued Medications*. Disclosure of HIV status occurred because of children’s consistent and endless questioning about the rationale for continued medications without disease symptoms and why they were the only ones taking daily medications, and their siblings and playmates were not going through similar experiences.

*Every time I met the healthcare provider*, *I pestered them to tell me why I was taking medication daily-*. *I insisted that I should be told the truth*., *I always asked*, *what health problem do I have*? *(Male informant; 15 years old; out of school*).

*I asked my mother why do I take medicine every day*? *I also asked her why I was the only one in the family taking that medicine (female informant; 17 years; out of school*).

*3*.*2*.*1*.*3 Refusal to take medications*. In other instances, disclosure occurred when some children had completely refused to continue taking the HIV medication unless they were told the reasons for doing so. Other children also pretended as if they were taking the tablets but would later throw them when out of sight as narrated by the following three informants:

*I refused to take the drugs [ARVs]they were giving me because I was not experiencing any pain until I was told the reasons (Male informant; 17 years old; in school*).

*I used to pretend as if I was taking the medicine*, *I would hold a cup of water*, *then get the tablet and throw it behind instead of swallowing (female informant; 16 years old; in school*).

*I deliberately refused to take it[ARVs] and……I refused to go to the health facility for refills (Female informant*, *17 years old; out of school*).

*3*.*2*.*1*.*4 Intermitted illnesses*. Intermittent illnesses such as chronic skin diseases and recurrent fevers were other circumstance that led to children receiving disclosure of their HIV status in the Masaka region.

*I used to fall sick recurrently I used to have on and off fever and every time I was staying in bed (male informant; 17 years; out of school*).

*I had started falling sick and I was physically looking terribly bad*. *I had wounds all over my body and I could not even put on clothes*. *When we went to the health center then they told us that I have HIV (male informant; 17 years; out of school*).

#### 3.2.2 Disclosure process

This theme explains how the disclosure of HIV status to children occurred in this study. The theme reports on the disclosure process as perceived by the children. Two subthemes are discussed namely: disclosure preparation and disclosure event

*3*.*2*.*2*.*1 Disclosure preparation*. During preparatory engagements about disclosure, children learn about their medical condition partially through providing them information on issues such as blood cells, disease particles and the immune system. In this study children reported that they were not told of any health related information relating to daily medications, were not assessed for any disclosure readiness nor prior disclosure counselling discussions with children. Instead, children were intimidated with death and high viral load outbursts as illustrated by the following informants:

*If you fail to adhere to the drugs*, *you will die because you have HIV with a high viral load (male informant; 15 years old; in school*).

*Mummy told me… if you do not take medicine*, *you will die…*. *you are sick with HIV infection (female participant; 17 years old; out of school*).

*Aunt told me*…*If you stop taking the medicine*, *you will die …*. *You got infected with HIV from your mother (male informant; 15 years old; out of school*).

*3*.*2*.*2*.*2 Disclosure event*. Children mentioned that the disclosure event was unexpected and happened accidentally. They also reported receiving minimal information about the HIV illness since the disclosure was unplanned and a one-time event, with no follow-up discussions regarding the disclosed results with children to monitor how well they were adjusting to the results or the impact of the disclosed information on their emotional wellbeing as reported by the following two informants:

*Mummy told that me that I have HIV*. *That is the only thing she told me (Male informant; 17 years old; in school*).

*I asked the health worker*, *what health problem I had*, *she replied*, *you got infected with HIV (male participant; 15 years old; out of school*).

*I asked mummy why my condition was not healing*? *She replied you were born with HIV (Female informant; 17 years; out of school*).

#### 3.2.3 Reactions to disclosure

The reactions to receipt of disclosure of HIV status varied among children. Two subthemes were identified namely: emotional distress and worries.

*3*.*2*.*3*.*1 Emotional distress*. The news of HIV diagnosis in these children elicited a lot of emotional distress and expressions of anxiety. Children reported being confused with feelings of general body weakness and nausea and vomiting as illustrated by the following excerpts:

*……*.*I got mentally confused and became depressed upon learning that I was HIV infected (male informant; 17 years; out of school*).

*I felt sick*, *the moment they told me that I have HIV……It haunts me even up to now*, *it is still haunting me and sometimes when I think about it*, *I get nausea and feel like vomiting (female informant; 17 years old; in school*).

*I cried and I even refused to take medicine for about three days (female informant; 17 years old*, *out of school*).

I failed to eat food for days just thinking about only that (female informant; 17 years old; out of school).

*3*.*2*.*3*.*2 Anger and irritation*. As a result of receiving news of an HIV diagnosis, children developed feelings of anger and irritation towards their mothers and their HIV uninfected siblings. They got annoyed of their mothers and felted irritated why only them and not any other sibling got infected with HIV.

The following informants narrate their story in these excerpts:

*After the health worker disclosing to me…*.*I got so annoyed with my mother for infecting me…*. *(Male participant; 17 years old; in school*).

*…*.*I felt so irritated inside me*. *Why me*? *Why me alone*? *My mother gave birth to seven children*, *all the other siblings are not infected(Male informant; 15 years; in school*).

*I felt angry… how come that I got infected with HIV among all other children that my mother produced*? *(Male informant; 17 years; out of school*).

*I became angry and even refused to take medicine [antiretroviral treatment] for three days (female participant; 17 years old; out school*).

#### 3.2.4 Post disclosure psychosocial experiences

All children in the sample described some form of post disclosure psychosocial experiences. Both negative and positive disclosure psychosocial- experiences were reported by the children. The positive disclosure psychosocial experiences included one subtheme: support from peers and community members while the negative disclosure psychosocial experiences included two subthemes: stigma and discrimination and insults and belittlements. The negative psychosocial experiences happened in school for school going children, in the community and in families for out of school children.

*3*.*2*.*4*.*1 Stigma and discrimination*. School going children experienced stigma and discrimination from school mates following their caregivers disclosing HIV status to school authorities to promote better ART adherence. This happened mainly for children in boarding schools. As a result, some school staff revealed children’s HIV status to other students and the cycle continued as illustrated by the following excerpt:

*At school*, *when students got to know my status……*, *they then started to discriminate me and had less associations with me for example the plate that I would use no other student would want to use it*, *whatever plate I touched they first washed it even if it was already clean*. *During that period*, *I felt fed up with the world (Male informant; 17 years old; in school*).

Out of school children reported being overwhelmed by the stigma and discrimination following disclosure inflicted on them by community members and their own HIV negative relatives, fathers, and siblings. One of the children narrated that her own father went to an extent of denying fathering her by saying that they cannot father an HIV infected child and did not want to be associated with her as explained by the following excerpt:

*There are relatives of mine who said*, *she deserves to die*. *One of them is my father… when he was told that I am HIV positive …unfortunately*, *he said that I am not his daughter (Female informant; 17 years old; out of school*)

In other cases, siblings and other family members dominated in stigmatizing these children as described by the following excerpts:

*When they were told that I am HIV positive*, *my two elder brothers started to discriminate me(male informant; 15 years old; in school*).

*I experienced stigma mostly from the families that were much related to my mother once they got to know about it [HIV status] (Male informant; 17 years old; in school*).

In the communities, there were also individuals who started discriminating children once they got to know about the children’s HIV status either by seeing them at the HIV clinics or after being told by third parties as illustrated by the following excerpt:

*Whenever* I *pass by the boda-boda men [commercial cyclists]*, *you hear them saying*, *that girl is already HIV positive with high viral load…*. *…she is a walking dead body*, *she is dead (Female informant; 17 years old; out of school*).

*3*.*2*.*4*.*2 Insults and belittlements*. Children reported getting insults and belittlements from community members after their caregivers requesting adult HIV patients with bicycles and motorcycles to offer lifts to children when going to pick their HIV medications at the facilities since all were getting their HIV care from same facilities as caregivers attended to other family responsibilities. These community members went on to tell other people, about the children’s HIV status, in the community and the cycle continued. As a result, children started getting unpleasant experiences such as being insulted and belittled as narrated by the following informants:

*My friends started belittling me after getting to know my HIV positive status*. *One of them insulted me by saying*, *‘do not familiarize me*! *go and take your drugs away from me’*. *Such things were an insult to me (male informant; 17 years old; out of school*).

*We were dancing at my uncle’s inauguration ceremony of his house and one of the adolescents came to me and I ignored him*. *He insulted me by saying*, *“why are you feeling proud when even you are infected with HIV……it hurt me (Female informant; 17 years old; in school*).

*3*.*2*.*4*.*3 Support from peers and community members*. Although majority of the children reported negative post disclosure psychosocial experiences, a few children reported positive post-disclosure psychosocial experiences including receiving moral and transport support to the facility by some community members to pick HIV medications and during child and adolescent clinic days by peers as illustrated by the following informants:

*They[community members] became my friends after knowing my [HIV*] *status They also show me that the status I am in was not my own making……*.*sometimes you may lack transport and they[community members] give you a means of transport to the facility for clinic visits(Male informant; 17 years; in school*).

*Every time I see other children[peers] coming to also pick their drugs*, *I get a feeling that I am not alone who take the drugs…I get strong encouragement (Male informant; 15 years old; in school*).

Also, for children who were in boarding school, positive post-disclosure experiences involved receiving support from school staff and friends to improve ART adherence and clinic appointment keeping as illustrated by the following informants:

*At school*, *the matron is responsible for us because it is where we keep our medicine*. *We take the medicine at the same time*, *and if you do not show up*, *the colleagues look for you until you go and take your medicine (female informant; 16 years old; in school*).

*At school four of my friends know about it [HIV status]*, *if I forget to take the medicine [antiretroviral treatment]*, *they remind me(male informant; 17 years; in school*).

*My mother told some teachers and the school nurse about my HIV status…one of them reminds me about clinic appointment dates*, *whenever I forget (male informant; 17 years; in school*).

#### 3.2.5 Self-disclosure to others

Children’s self-disclosure of their HIV status to others attracted both positive and negative perceptions and experiences. There were three subthemes that emerged from this theme of self-disclosure to others. One subtheme was negative namely concerns of confidentiality and two positive subthemes viz: reminders about medication time and maintaining sexual relationships.

*3*.*2*.*5*.*1 Negatives of disclosure*. Some children, due to fears and concerns of not keeping the disclosed results confidential, preferred to conceal their HIV status from other people including friends, close associates, and relatives. Thus, many children kept their HIV status to themselves as a secret as illustrated by the following informants:

*I ensure that I hide it[HIV status] from them [community]*. *This is because someone might know about it and he or she starts telling the whole village so*, *I must keep it confidential (female informants; 17 years old; out of school*).

*My siblings do not know that I have HIV and I can longer stay with them for some good time because they will know it (female informant; 17years old; out of school*).

*I do not want other people to know*. *If I disclose to other people*, *they will spread rumours and not keep it [HIV status] confidential (female participant; 17 years old; out of school*).

*3*.*2*.*5*.*2 Reminders about medication time*. Some children disclosed their HIV status to teachers and superiors because these categories of people were trusted. This self-disclosure resulted into positive experiences. For example, children were supported in constantly being reminded to take their medications timely at the workplace for working children and at school for school-going children. School going children in boarding schools had their bags ring-fenced by school authorities from interference by other students in the dormitories as illustrated by the following excerpts:

I disclosed to the headteacher and another teacher who is a friend to me to help ring-

*fence my suitcase from being opened by students and remind me about medication time*. *The headteacher used to come for me once it is medication time even when the teacher was still in class and call me out to tell me that it is medication time (male informant; 17 years; in school*).

*I shared my HIV status with my boss before she did*. *She also takes medicine*, *so we could not fear each other because she used to remind me*. *When it is 08*:*00 pm*, *I take my medicine*. *My boss also reminds me because for her she takes it in the morning*. *We remind each other (female informant; 17 years old; out of school*).

*3*.*2*.*5*.*3 Sexual relationships*. A few children described sexual relationships as a safe haven for self-disclosure because they perceived that disclosure would strengthen these relationships as described by the following informants:.

*I disclosed my HIV positive status before we got into this relationship*. *He replied*, *it is ok*. *He is HIV negative*, *but instead he accepted to have our relationship move on (female participant; 17 years old; out of school)*.

*My husband is aware*. *He has no problem*. *Sometimes he escorts me while going to the health facility for my clinical appointments(married female participant; 17 years old; out of school*).

*There are some adolescents who are my friends; they wanted to have a sexual relationship with me*, *and I told them the truth that I have HIV because for sure they were my friends*. *This did not affect my relationship with them because we continued getting along (female informant; 17 years old; out of school*).

## 4 Discussion

The main aim of this research was to explore the experiences of children regarding disclosure of their HIV status. Key findings revealed that children were not prepared to receive disclosure and disclosure occurred as a one-time event and not as a process irrespective of the discloser. This finding of disclosing to children as a one-time event without prior preparations or follow-up discussions on disclosure contradicts Uganda national disclosure guidelines [34] and standardized international guidelines for providing disclosure to children as a process and not as an event [48–51]. However, similar findings have been reported in Ghana [52] where it was also revealed that disclosure to children aged 9–19 years was done with little or no preparation because of children persistently questioning the rationale for continued medications, refusal to take medications or poor adherence to ART. This means that there is need to develop a structured pre-and post- disclosure process to facilitate children to accept their new status. A study done in Thailand [53] suggests a four-step provider-assisted and counselling-based child disclosure model including eligibility screening and invitation, readiness assessment and preparation for disclosure, provider-assisted disclosure session, and post-disclosure follow-ups. This is in line with standard recommendations [48, 54] and within the socio-cultural context of children for disclosure to result into positive emotional outcomes. Our data suggest urgent disclosure training needs for HCPs to provide process-oriented disclosure and to cascade these skills to caregivers of HIV infected children appropriately.

The results of persistent questioning for the rationale for continued medications and poor adherence to ART among children are also consistent with recent findings from Ghana in which children aged between 5 and 18 years recurrent questioning of caregivers about continuous medication intake increased the odds of HIV disclosure [55]; and findings from Zambia in which disclosure of HIV status was dictated by repeated and persistent inquiries by children regarding reasons for taking daily medication despite not being sick [56]. Similarly, in China, poor adherence to HIV treatment by children was among the key reasons why they were told their HIV status [27]. The results from this study suggest that disclosure decisions should be contextualized and distinctively shaped by the socio-cultural setting of each child.

It is clear from the findings that children reacted to the disclosure events with distress, anger and irritation. These emotional reactions observed in this study are consistent with a South African study, in which HIV positive adolescents also experienced negative emotions such as emotional upset, confusion, shock, and distress [30]. On the other hand, these negative emotional reactions mean that although children were developmentally mature, by Piagetian standards [57], to comprehend concepts of the HIV illness [58, 59], were never assessed for disclosure readiness and were unprepared to receive their HIV status. Early and focused disclosure preparation of children should be done to avoid such negative experiences on their self-image.

There were also psychosocial experiences that resulted from the disclosure including stigma and discrimination. Stigma and discrimination were inflicted to children during schooling, in the community and by their own HIV negative relatives, fathers, and siblings after these third parties gained knowledge of children’s HIV status. These findings are in tandem with recent findings from Uganda [60] in which children described unfair and harsh treatment by their HIV negative siblings and adults that often led to negative emotions including embarrassment and emotional pain leading to isolation to avoid being hurt. In school settings, this was often preceded by rumors and gossiping about them[HIV infected children] through pointing fingers, teasing and frequent laughing [61]. This suggests that interventions to manage stigma and discrimination in the context of the social worlds of children, community, school settings and family environments are needed to better the wellbeing of children as they navigate with managing HIV chronicity in their lives.

However, children who received moral support from other adolescents through sharing HIV status disclosure testimonies during adolescent clinic days and boarding schooling times revealed the importance of peer support. This is consistent with findings from a one systematic review [62] which also revealed the usefulness of peer support in improving children wellbeing through supporting post disclosure psychosocial processes by providing safe spaces. It appears that disclosure of HIV status defines sociocultural community identities and generates new forms of citizenship [63] shaped by group concerns [64]. However, these findings differ from an urban Ugandan Study [22] in which children coped with the HIV status disclosure through concealment, spirituality/religiosity, and avoidance. These differences may be due to differences in study setting since this study was conducted in a rural environment while that of Mutumba and colleagues [22] was conducted in Kampala and suggest the need for different disclosure interventions for different socio-cultural environments.

On self-disclosure of their HIV status to others, children preferred to keep this a secret due to concerns of stigma and confidentiality in the community. This finding is consistent with findings from South Africa where adolescents who were perinatally infected with HIV maintained secrecy to be accepted by their peers and protect themselves from stigma and isolation [30, 65]. Mpofu and Jacobs [66] found that children feared self-disclosure to peers because of being gossiped at or laughed at in the community and at school. In a recent Ugandan study [61], children described several instances where other people rumored, gossiped, pointed fingers at them and made them a laughingstock. These findings suggest a need for ongoing psychosocial post-disclosure support interventions for children particularly in rural settings where psychosocial support services are insufficient to address their emotional wellbeing. However, other children who self-disclosed to friends, superiors and sexual partners benefitted. They received moral and financial support including support from community members. These mixed findings are consistent with previous studies [22, 29, 67] and suggest the uniqueness of the disclosure experiences requiring interventions that consider socio-cultural contexts in design.

### 4.1 Study limitations

This analysis was based on in-depth interviews of HIV infected children taking daily ART. A combination of in-depth interviews and focus-group discussions would have provided more information to corroborate the findings. Secondly, the results were based on experiences of children which are prone to recall bias and social desirability. However, our findings reveal important findings that inform the need for policy and practice interventions to improve the quality of disclosure of HIV status to children either by caregivers or HCPs.

### 4.2 Practice implications of the study

The findings of this study have shown clearly that disclosure of HIV status to children occurred as a one-time event, with neither preparatory nor follow-up meaningful disclosure support discussions with children. Thus, disclosure trainings for disclosers during pre-disclosure to assess psychosocial risks and post disclosure evaluation, to document disclosure outcome, in a socio-culturally contextualized manner, using a process-oriented disclosure framework is urgently needed. It is also apparent that the skills of healthcare providers in psychosocial support, such as psychologists and related professionals, need to be harnessed to continually support children with emotional wounds resulting from haphazard disclosure of HIV status.

### 4.3 Conclusions

This study revealed that disclosure of HIV status to children was happening as a one-time event with no pre-disclosure preparatory planning or focused post-disclosure counselling follow-up to help children cope with their HIV status in the context of HIV chronicity. HIV disclosure should be planned and progressively provided before children start to ask too many questions, show poor adherence, or refuse to take ART medications. This may be done through training and mentoring healthcare providers and caregivers in this setting using disclosure tool kits [49–51] and disclosure guidelines [34, 48] that emphasize careful preparatory disclosure planning and post-disclosure follow-up counselling based on a process-oriented framework.
